# High-dose dietary zinc oxide mitigates infection with transmissible gastroenteritis virus in piglets

**DOI:** 10.1186/1746-6148-10-75

**Published:** 2014-03-28

**Authors:** Weidong Chai, Silke S Zakrzewski, Dorothee Günzel, Robert Pieper, Zhenya Wang, Sven Twardziok, Pawel Janczyk, Nikolaus Osterrieder, Michael Burwinkel

**Affiliations:** 1 Institut für Virologie, Freie Universität Berlin, Berlin, Germany; 2 Institut für Klinische Physiologie, Charité - Universitätsmedizin Berlin, Berlin, Germany; 3 Institut für Tierernährung, Freie Universität Berlin, Berlin, Germany; 4 Molekularbiologie und Bioinformatik, Charité - Universitätsmedizin Berlin, Berlin, Germany; 5 Bundesinstitut für Risikobewertung, Abteilung für Biologische Sicherheit, Fachgruppe für Molekulare Diagnostik und Genetik, Berlin, Germany

**Keywords:** Zinc oxide, Coronavirus, Transmissible gastroenteritis virus, Cytokine, Morphometry, Electrophysiology, Zinc transporters

## Abstract

**Background:**

Zinc (Zn) supplementation has been shown to reduce the incidence of diarrhea and to protect animals from intestinal diseases, but the mechanisms of this protective effect against virus infection *in vivo* have not yet been elucidated. Transmissible gastroenteritis virus (TGEV) causes diarrhea in piglets with an age-dependent decrease of severity.

**Results:**

We used 60 weaned piglets that were divided into three groups to evaluate the effect of different Zn levels added to a conventional diet (50 mg Zn/kg diet, Zn^low^, control group). The other groups received the diet supplemented with ZnO at final concentrations of 150 mg Zn/kg diet (Zn^med^), or 2,500 mg/kg diet (Zn^high^). Oral challenge infection with TGEV was performed when the pigs had been fed for 1 week with the respective diet. Half of the piglets of each group were sacrificed at day 1 and 18 after challenge infection. Fecal consistency was improved and body weights increased in the Zn^high^ group when compared to the other groups, but no direct effect of Zn concentrations in the diet on fecal TGEV shedding and mucosal immune responses was detectable. However, in the Zn^high^ group, we found a prevention of villus atrophy and decreased caspase-3-mediated apoptosis of jejunal epithelium. Furthermore, pigs receiving high Zn diet showed a down-regulation of interferon (*IFN*)-*α*, oligoadenylate synthetase (*OAS*), Zn transporter SLC39A4 (*ZIP4*), but up-regulation of metallothionein-1 (*MT1*), as well as the Zn transporters SLC30A1 (*ZnT1*) and SLC30A5 (*ZnT5*). In addition, forskolin-induced chloride secretion and epithelial resistance were controlled at a physiological level in the Zn^high^ but not the other groups. Finally, in the Zn^high^ group, we documented an earlier and higher systemic TGEV-specific serum antibody response.

**Conclusions:**

These results suggest that high dietary Zn could provide enhanced protection in the intestinal tract and stimulate the systemic humoral immune response against TGEV infection.

## Background

Several *in vitro* studies have shown that zinc (Zn) has broad-spectrum antiviral activity against a variety of viruses, such as human immunodeficiency virus, transmissible gastroenteritis virus (TGEV), equine arteritis virus, and severe acute respiratory syndrome coronavirus [1-6]. Many potential mechanisms have been suggested to explain the potential beneficial effect of Zn against virus infections. For example, Zn mediates antiviral effects through the inhibition of nidovirus RNA-dependent RNA polymerases or other proteins essential for the different phases of the viral life cycle [5,6]. In addition, Zn participates in initiating and maintaining robust immune responses, in particular cytokine production and modulation of the activity of immune cells [7]. Zn induces the production of innate interferon (IFN)-α and also immune IFN-γ, and can potentiate the antiviral action of IFN-α, but not of IFN-γ [8]. Clearance of viral infections requires cytotoxic T lymphocytes, which are also highly dependent on the presence of Zn [7]. Antibody production during both the first and an immunological memory response is disturbed by Zn deficiency [9,10], indicating that Zn is necessary for optimal results following vaccination.

In swine nutrition, especially in the North American swine industry, high levels of Zn oxide (ZnO, 2,000-3,000 ppm) are often added to the diet of weaned pigs, since such addition was shown to reduce non-specific post-weaning diarrhea and improve performance in this critical period of dietary change [11-13]. Diarrhea is caused by impaired intestinal epithelial barrier function, which most likely leads to malnutrition and decreased uptake of micronutrients, including Zn. It was shown that oral Zn supplementation with high doses was able to counteract this loss, improve intestinal mucosal integrity as well as absorption of water and electrolytes [12,14]. Furthermore, it leads to a faster regeneration of the gut epithelium [15]. However, because of environmental concerns, the maximum level of Zn allowed in pig diets was set up to 150 ppm in the European Union, irrespective of the Zn formulation [16].

Zn homeostasis is maintained in the body through a variety of transporters and Zn binding proteins [17]. High levels of dietary Zn provided as ZnO have been recently shown to outbalance Zn homeostasis with increased accumulation of Zn in various organs including the small intestine of piglets [18,19]. Since intestinal Zn uptake can also take place through passive diffusion, it is likely that very high dietary Zn levels would indirectly increase the intestinal barrier function as a protection mechanism of the epithelium. In addition, metallothionein that is induced by Zn accumulation in intestinal tissue may also protect the tissue from oxidative damage.

Due to suboptimal immune functions, newborn as well as weaned piglets are particularly susceptible to infection by various pathogens, among them TGEV, which causes severe to mild gastroenteritis in piglets, depending on the age [20,21]. Our previous study [5] showed that high Zn levels markedly reduced TGEV titers as well as viral RNA and protein synthesis *in vitro*, but there is no report about antiviral effects of Zn supplementation in pigs. The aim of this study was to close the knowledge gap and evaluate the antiviral potential and possible protection mechanisms of increased dietary Zn supplementation against TGEV infection in weaned piglets.

## Results

### High-dose Zn prevents diarrhea in piglets but does not affect other trace elements and virus shedding

**Figure 1:**
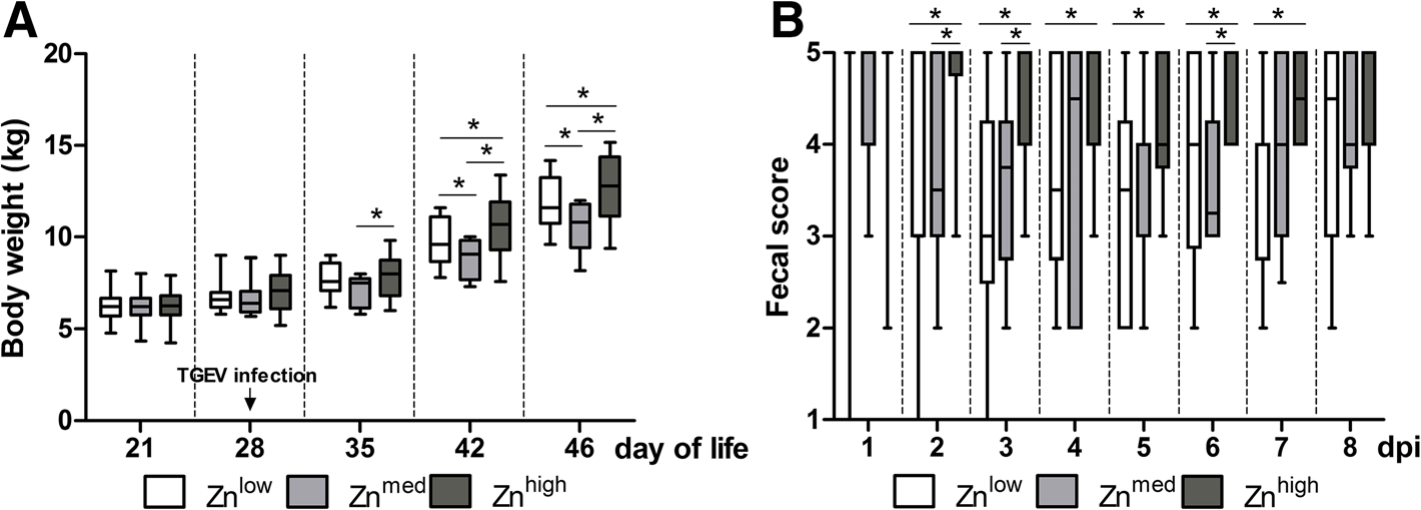
**Body weight and fecal score of TGEV-infected piglets.** Piglets fed with Zn^low^, Zn^med^ and Zn^high^ were orally infected with TGEV. **(A)** Body weight was recorded at given time points and **(B)** fecal scores (from 1 to 5, where 1 means watery and 5 hard and dry stool) were recorded daily after infection. Boxes indicate medians (n = 10) (horizontal lines) and the lower and upper quartiles (bottoms and tops of the boxes). The vertical bars in the box plots indicate the minimal and maximal values recorded. Asterisk indicates statistically significant difference (p ≤ 0.05) between the groups.

TGEV infection caused only mild symptoms and there was no difference in dehydration, anorexia, lethargy and body temperature when the different Zn treatment groups were compared. However, the body weight in Zn^high^ was higher at 7, 14 and 18 dpi in comparison to both other groups (Figure 1A). Furthermore, the fecal score from 2 to 7 dpi was also higher in Zn^high^ compared to the Zn^low^ control group (Figure 1B).

Serum and liver Zn concentrations were higher in the Zn^high^ group as compared to the Zn^med^ and Zn^low^ groups, but other trace elements were not affected. In addition, there was an increased Zn, manganese and iron concentration, but decreased copper concentration, in both liver and serum from 1 to 18 dpi (Additional file 1: Table S1).

Low amounts of shedding TGEV could be detected by qPCR from 1 to 6 dpi, irrespective of Zn feeding group. The highest incidence was observed at 4 dpi with 4 out of 10 positive TGEV shedding piglets in each group.

### Systemic and mucosal immune responses

**Figure 2:**
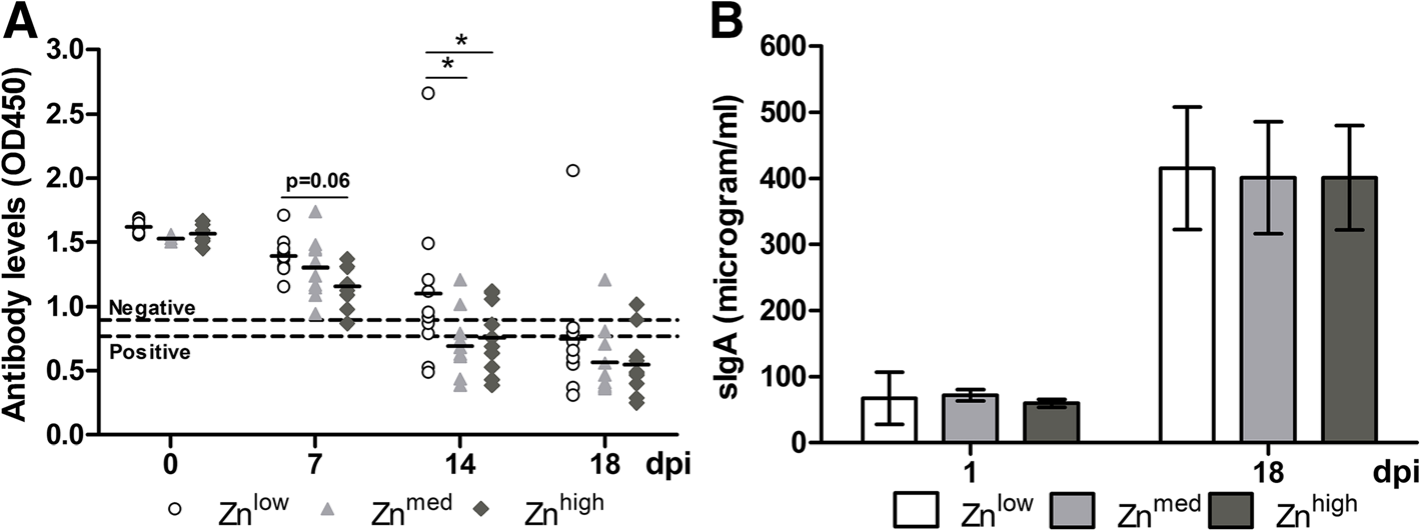
**Development of systemic and mucosal immune responses after TGEV infection. (A)** TGEV-specific antibodies measured by competitive ELISA are shown as optical densities (OD), measured at 450 nm, from 0 to 18 dpi. An OD_450_ value higher than 0.9 was considered negative (upper dashed line), an OD_450_ value lower than 0.77 was considered positive (lower dashed line), and an OD_450_ value between 0.9 and 0.77 was considered questionable. **(B)** The concentration of sIgA in intestinal wash fluid was measured by a direct ELISA. Results are shown as the mean value ± standard deviation. Asterisk indicates statistically significant difference (*P* ≤ 0.05) between the groups.

Figure 2 shows the systemic and mucosal immune responses of piglets as measured by ELISA. At the time of challenge, all piglets fed with different concentrations of Zn were negative for TGEV-specific serum IgG antibodies. The serum antibody response after infection occurred earlier in Zn^high^ piglets and was measurable already at 7 dpi, when all piglets from this group tended to develop higher (*P* = 0.06) TGEV-specific serum antibody titers than piglets in the Zn^low^ group. At 11 dpi, seroconversion was clearly detectable in almost all animals. Greater antibody levels were detected in Zn^med^ (*P* = 0.009) and Zn^high^ (*P* = 0.03) groups compared to the Zn^low^ group at 14 dpi (Figure 2A).

The levels of sIgA antibodies in intestinal wash fluids were increased 6 to 7-fold from 1 to 18 dpi, showing that mucosal adaptive humoral immune response had also been induced after infection, but the differences between the dietary groups were only marginal (Figure 2B).

### Gene expression profiles

**Figure 3:**
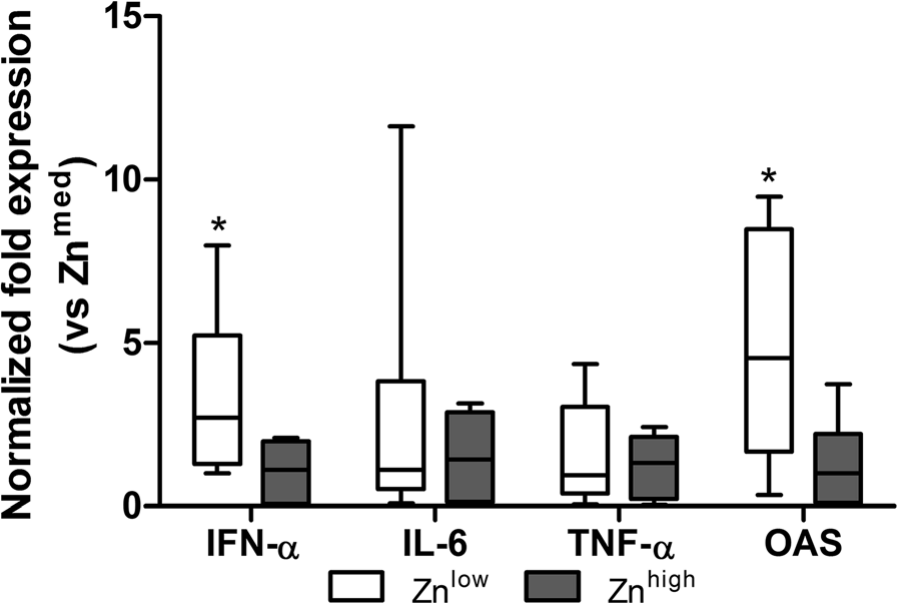
**Cytokine expression in intestinal tissues of Zn-treated piglets at 1 dpi.** The expression of selected cytokines was assessed by quantitative RT-PCR. The expression of *IFN-α* and *OAS* was significantly increased in the Zn^low^ compared to the Zn^high^ group. Boxes indicate medians (n = 10) (horizontal lines) and the lower and upper quartiles (bottoms and tops of boxes). The vertical bars in the box plots indicate the minimal and maximal values recorded. Asterisk indicates statistically significant differences (*P* ≤ 0.05) between Zn treatment groups.

To further elucidate the potential effect of Zn supplementation on the immune response and on Zn transport, we examined the expression of genes for cytokines and metal binding/transport proteins in intestinal tissues in the three Zn treatment groups. There was a statistically significant increase of *IFN-α* expression in the Zn^low^ group compared to Zn^high^ group (*P* = 0.009). 2′, 5′-oligoadenylate synthetase (*OAS*) is one of IFN-stimulated gene products (ISGs), and there was a decreased expression of this enzyme in the Zn^low^ group (*P* = 0.01) (Figure 3). Expression of *ZIP4* was higher and *ZnT1*, *ZnT5* and *MT1* were lower in Zn^high^ group compared with two other groups (Table 1). Expression of *IL-6*, *TNF-α* and *ZnT2* did not differ between treatments.

**Table 1:** **Mean relative gene expression of zinc transporters and metallothionein in jejunal tissue of piglets at 1 and 18 dpi**^**1**^

**Gene**	**1 dpi**			**18 dpi**			**Significance**		
	**Zn**^**low**^	**Zn**^**med**^	**Zn**^**high**^	**Zn**^**low**^	**Zn**^**med**^	**Zn**^**high**^	**Diet**	**Time**	**Diet × Time**
*ZIP4*	1,22 ± 0,16	1,46 ± 0,16	0,44 ± 0,08	3,91 ± 0,44	1,68 ± 0,295	0,39 ± 0,18	***	**	**
*ZnT1*	0,94 ± 0,11	0,81 ± 0,08	1,47 ± 0,35	0,63 ± 0,06	0,77 ± 0,075	1,38 ± 0,23	**	ns	ns
*ZnT2*	1,39 ± 0,44	0,50 ± 0,14	1,82 ± 0,54	1,10 ± 0,58	1,09 ± 0,634	0,85 ± 0,40	ns	ns	ns
*ZnT5*	0,68 ± 0,06	0,61 ± 0,10	0,86 ± 0,13	0,72 ± 0,10	0,50 ± 0,07	1,04 ± 0,14	**	ns	ns
*MT1*	0,12 ± 0,02	0,12 ± 0,01	2,59 ± 4,23	0,10 ± 0,01	0,15 ± 0,031	11,71 ± 4,28	***	ns	ns

Data are means ± SEs, n = 10/group. Significances are depicted as: **, P < 0.01; ***, P < 0.001.

^1^Abbreviations: *MT1* metallothionein-1, *ZIP4* Zn transporter *SLC39A4, ZnT1* Zn transporter *SLC30A1*, *ZnT2* Zn transporter *SLC30A2*, *ZnT5* Zn transporter *SLC30A5*.

### Histology and immunohistochemistry

**Figure 4:**
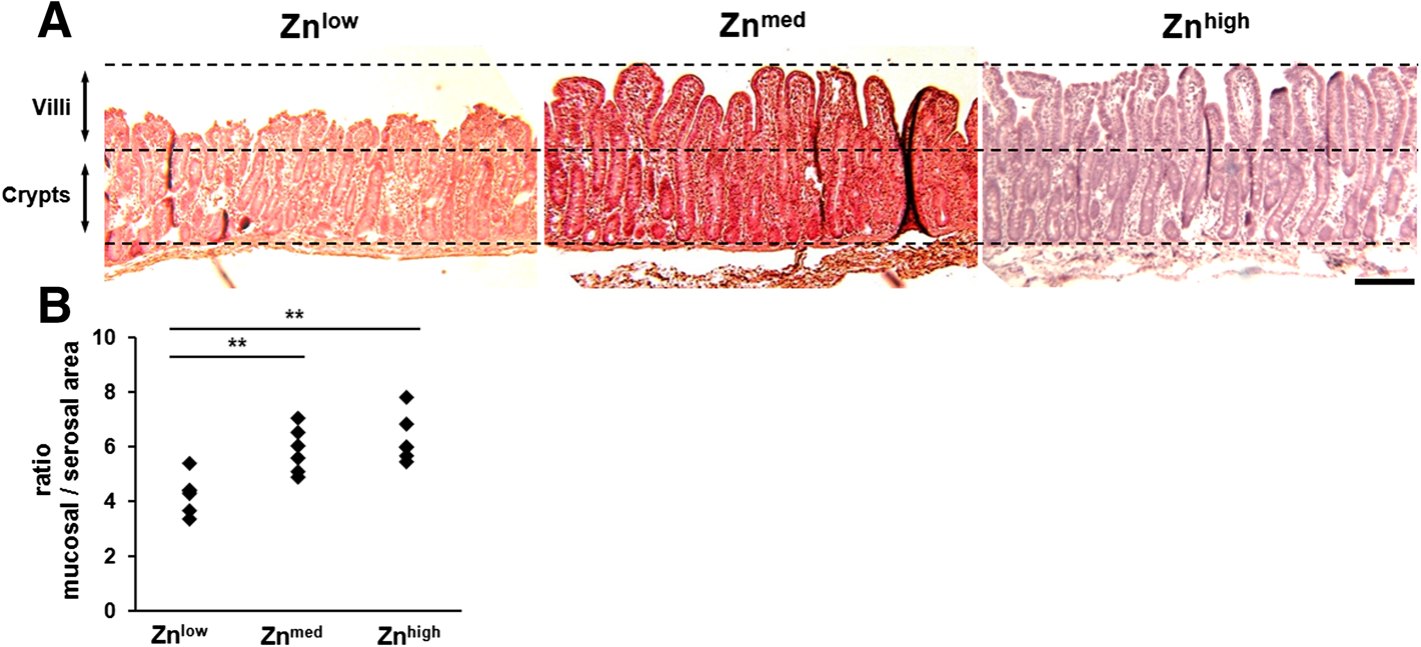
**Morphometrical analysis of jejunal tissue at 1 dpi. (A)** Jejunal tissue from piglets fed with Zn^low^, Zn^med^, and Zn^high^ diets, respectively, were sliced and H&E stained. The bottom and upper dashed lines indicate the basal and apical borders of the epithelium. The middle dashed line represents the transition zone from where crypts go down and villi go up. TGEV infection in Zn^low^ piglets resulted in villus atrophy, which could be prevented by Zn^med^ and Zn^high^ diets. Scale bar = 200 μm. **(B)** H&E-stained jejunal specimens were morphometrically analyzed by measuring the lengths of apical epithelial and the mucosa’s muscular linings. The ratio of mucosal-to-serosal surface area represents a measurement for the effective epithelial area, which was significantly decreased under Zn^low^ (*P* ≤ 0.01, n =6) compared to Zn^med^ and Zn^high^ (n = 6 each). The latter conditions were not significantly different.

**Figure 5:**
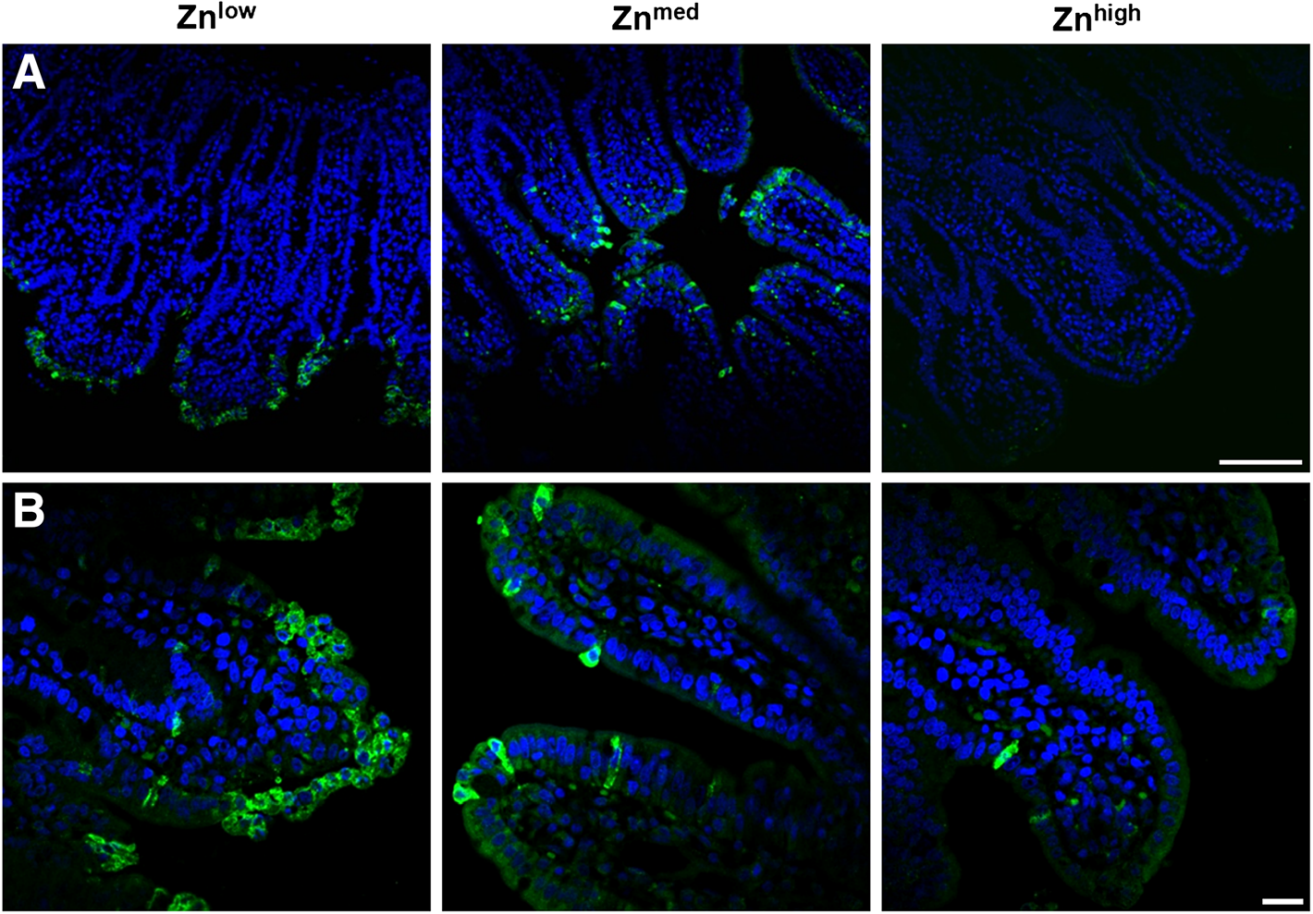
**Immunofluorescence staining of caspase-3 in jejunal epithelium at 1 dpi.** Apoptotic cells are presented by cells being positive for cleaved caspase-3 (as depicted in green). Nuclei (DAPI staining) are presented in blue. **(A)** When the jejunal epithelium was affected by TGEV infection, only the villus lining was apoptotic, while the crypt epithelium stayed intact. Scale bar = 100 μm. **(B)** Under Zn^low^ diet, most of jejunal enterocytes were cleaved caspase-3-positive and their shape was distorted, whereas under Zn^med^ treatment, the amount of cleaved caspase-3 signals was reduced and the cell shape was not affected. Under Zn^high^, apoptotic cells were extremely rare and cell morphology was not impaired. Scale bar = 20 μm.

To further investigate the effect of Zn supplementation on TGEV infection in piglets, histological changes in intestinal tissues of piglets were examined. Piglets from the Zn^low^ group showed a destruction of the architecture of intestinal tissue with villous atrophy, which resulted in a significant reduction of the intestinal surface area by a factor of 1.38 (n = 6, *P* = 0.009) compared to the Zn^med^ group where no villus atrophy was observed (n = 6) (Figure 4A and B). No further changes in villus morphology in the Zn^high^ group (n = 6) could be detected (Figure 4A and B). In piglets from the Zn^low^ group, the majority of jejunal enterocytes was caspase-3-positive while being morphologically altered, whereas in Zn^med^ jejunal tissue the number of caspase-3-positive cells were drastically reduced and there was a further reduction of apoptotic cells in Zn^high^ animals (Figure 5A and B). The epithelial architecture of Zn^med^ and Zn^high^ jejunum was apparently not impaired. The crypt epithelium was not affected by TGEV infection at any Zn concentration.

### Intestinal epithelial resistance, paracellular permeability, and active transport

**Figure 6:**
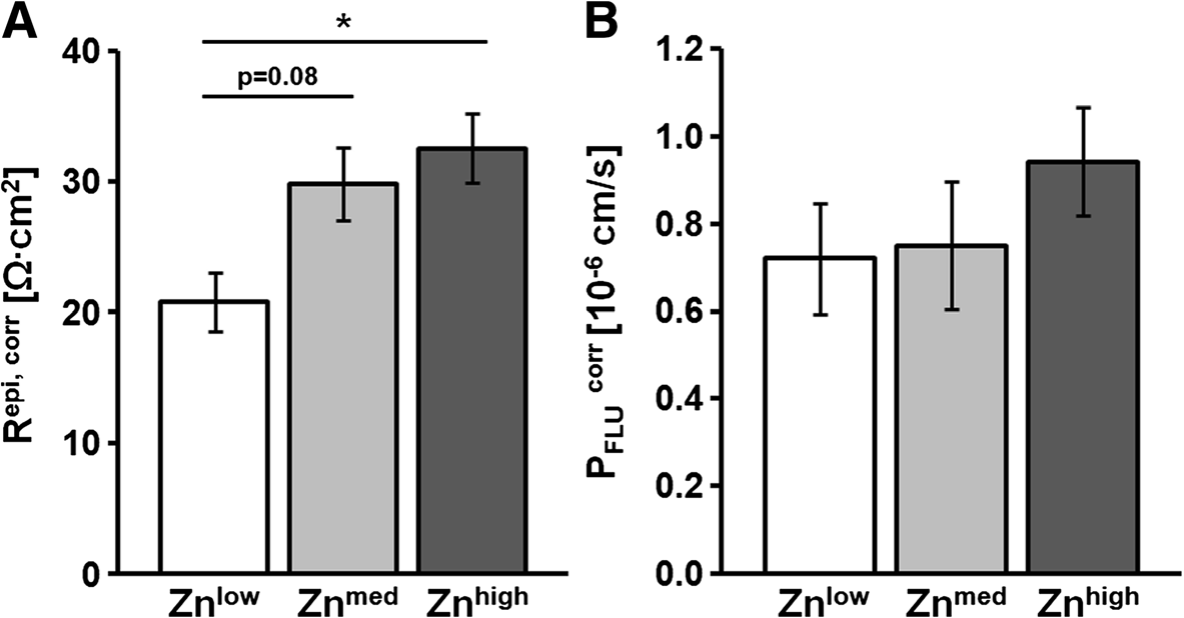
**Epithelial resistance and paracellular permeability at 1 dpi.** All values were corrected for the epithelial surface area (^corr^). **(A)** Epithelia from the Zn^low^ group exhibited significantly decreased R^epi, corr^ values (*P* = 0.01, n = 8) compared to the Zn^high^ group (n = 10) and a trend to values lower than those of the Zn^med^ group (*P* = 0.08, n = 10). **(B)** Fluorescein permeability (P_FLU_^corr^) did not significantly differ between Zn groups.

**Figure 7:**
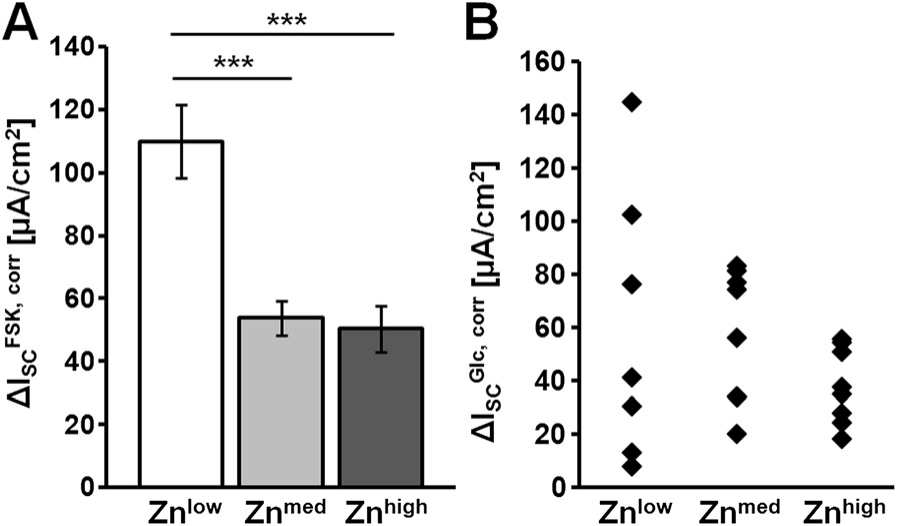
**Active transport of jejunal epithelium at 1 dpi.** All values were corrected for the epithelial surface area (^corr^). **(A)** Stimulation by forskolin (FSK) resulted in a significantly increased chloride secretory response (∆I_SC_^FSK, corr^) in the Zn^low^ group (*P* ≤ 0.001, n = 8) when compared to the Zn^med^ (n = 9) and Zn^high^ groups (n = 10). **(B)** Glucose-induced short-circuit currents (∆I_SC_^Glc, corr^) of Zn^low^ (n = 7), Zn^med^ (n = 8), and Zn^high^ (n = 8) were not significantly different. Note the big spread of individual values in the Zn^low^ group.

Taking into account the changes in the surface area (^corr^), R^epi, corr^, as a measure of epithelial integrity, was lower in tissues from the Zn^low^ group (20.8 ± 2.2 Ω•cm^2^, n = 8), compared to Zn^med^ (29.8 ± 2.8 Ω•cm^2^, n = 10; Zn^low^ vs. Zn^med^, *P* = 0.08) and Zn^high^ (31.4 ± 2.7 Ω•cm^2^, n = 10; Zn^low^ vs. Zn^high^, *P* = 0.01) (Figure 6A). Surprisingly, permeability to the paracellular marker fluorescein did not significantly differ in TGEV-challenged jejunal epithelia in any of the animals (Figure 6B). As an *ex vivo* measure for *in vivo* diarrhea susceptibility, forskolin (FSK)-induced chloride secretion was quantified as increase in short-circuit current, ΔI_SC_^FSK,corr^. It was found to be increased in jejunal tissue from TGEV-infected Zn^low^ piglets (ΔI_SC_^FSK, corr^, 110 ± 11 μA/cm^2^, n = 8) compared to Zn^med^ (54 ± 5 μA/cm^2^, Zn^low^ vs. Zn^med^, *P* = 0.0002, n = 9) and Zn^high^ (50 ± 7 μA/cm^2^, Zn^low^ vs. Zn^high^, *P* < 0.0001, n = 10) (Figure 7A), whereas ΔI_SC_^FSK, corr^ of Zn^med^ and Zn^high^ did not significantly differ. In contrast, average glucose-induced short-circuit currents (ΔI_SC_^Glc, corr^) were not different in jejunal tissues from piglets fed the tested Zn concentrations, however, against expectations, the range of ΔI_SC_^Glc, corr^ values was greatly increased in Zn^low^ compared to the other two conditions (Figure 7B).

## Discussion

High doses of ZnO (2,000 – 3,000 mg ZnO/kg diet) added to the diets of newly-weaned piglets were shown to improve performance and to reduce the occurrence of unspecific diarrhea [13]. TGEV infection causes villus atrophy with severe and frequently fatal diarrhea in newborn pigs, while the clinical signs in older piglets or in adult pigs are milder or inapparent because of a higher replacement rate of enterocytes compared to newborn piglet [22-24]. However, asymptomatic older piglets may serve as carriers, and the high mutation rates of coronavirus genomes may lead to the generation of more virulent genotypes. For this study we infected weaned piglets with a cell-culture adapted TGEV strain, for which direct antiviral effects of Zn were proven *in vitro*[5], and tested if direct antiviral and/or systemic effects of different Zn levels could be observed *in vivo*. Critical factors for the reduction in the mortality and morbidity of piglets from TGE include a reduction in the infectious agent, and oral rehydration therapy for the treatment of dehydration and metabolic acidosis associated with acute diarrhea [12].

In this study, TGEV infection caused only mild clinical symptoms, which can be explained by the piglets’ age and by the use of a tissue-culture adapted virus strain, which was chosen on purpose in order to directly compare results from this study with previous *in vitro* results [25]. Furthermore, in our study the piglets were provided with a relatively comfortable environment to minimize stress other than that induced by infection. It should be stressed that our experimental conditions vary substantially from those in commercial farming conditions. This could also contribute to the absence of severe clinical signs after infection. However, this study demonstrated that feeding the Zn^high^ diet improved the fecal score and led to higher body weights after infection in comparison to the Zn^med^ and Zn^low^ groups. Several studies demonstrated that feeding high levels of Zn reduces the incidence and severity of diarrhea and improved fecal consistency [26,27]. Our results are consistent with these findings showing that the Zn^high^ group had higher fecal scores compared to the Zn^med^ and Zn^low^ groups after infection. There was also a direct correlation between Zn levels and histological changes. TGEV infection in the Zn^low^ group led to destruction of the enterocytes of jejunal villi as reflected by marked villus atrophy. It has been reported that Zn plays a fundamental role in maintaining epithelial barrier integrity and function. For example, supplementation of Zn reduced methotrexate-induced intestinal damage and resulted in faster recovery [28], while it reduced intestinal permeability in Bangladeshi children with acute diarrhea and persistent diarrhea syndrome [29]. Furthermore, feeding supplemental Zn to rats with experimental colitis improved mucosal repair by regulating tight junction permeability [30]. In agreement with these data, higher R^epi, corr^ values in the Zn^med^ and Zn^high^ group indicate that Zn may prevent epithelial barrier loss induced by TGEV. The dramatic increase in ∆I_SC_^FSK, corr^ observed in the Zn^low^ group despite the reduced surface area may be interpreted as a protective mechanism. Chloride secretion is the basis of secretory diarrhea and might be regarded as a mechanism to rapidly extrude pathogens. The increase in ∆I_SC_^Glc, corr^ observed in some of the animals from the Zn^low^ group may be a compensatory effect and is in agreement with the observation that, even in the Zn^low^ group, animals gained weight at a normal rate. This considerably larger variance in the Zn^low^ group indicates that some animals were able to compensate reduced glucose uptake due to the loss of villi by increasing glucose transport capacity.

Zn is also important for the production of antibodies against intestinal pathogens [31]. In agreement with such finding, TGEV-specific serum antibody titers were detected earlier and at higher levels in Zn^high^ when compared to Zn^low^ piglets. This finding may be the result of multiple effects of Zn on antigen-presenting cells, T-cells and antibody-producing B-lymphocytes. IgA is the primary immunoglobulin isotype induced at the mucosal surface. Secretory IgA (sIgA) in mucosal secretions provides protection against bacterial and viral pathogens and neutralizes microbial toxins [32]. Zn can influence sIgA levels by altering the cytokine profile of stimulated immune cells residing in the gut-associated lymphatic tissue (GALT) [33]. Furthermore, sIgA responses are mediated through activated Th2 cells producing, among other, abundant amount of IL-6 [34]. In this study, there was an increased sIgA levels from 1 to 18 dpi, indicating that adaptive mucosal immune responses were induced by TGEV infection but not influenced by the diet. This finding is in accordance with results from Broom and colleagues [33], who also showed only slight differences of intestinal IgA concentrations between animals either receiving low or high levels of dietary ZnO. In accordance with these findings, the expression of *IL-6* in the intestinal tissues also showed no difference between the dietary groups at 1 dpi. Therefore, it is questionable if Zn plays a role in enhancing intestinal IgA concentration.

TGEV infection in piglets is characterized by a robust and early IFN-α production in intestinal secretions and in other organs [35,36]. At 1 dpi, higher *IFN-α* gene expression levels in the Zn^low^ group may be consistent with a more severe TGEV infection or higher virus loads in this group compared to the Zn^high^ group. The activation of *OAS*, one of the IFN-stimulated gene products (ISGs), can lead to apoptosis [37], potentially by indirectly triggering cleavage of caspase-3 [38]. The increased *OAS* level in the Zn^low^ group may reflect TGEV-induced apoptosis in intestinal epithelial, as it was shown, that the amount of caspase-3-positive cells was markedly increased in the Zn^low^ compared with the Zn^med^ and Zn^high^ group. This finding is in agreement with the histological observations: the high-grade villus atrophy in the Zn^low^ and normal jejunal mucosal morphology in the Zn^high^ group.

Metallothionein is known to be induced by exposure to heavy metal cations [39], and in line with a former study [18], the expression level of *MT1* was higher with concomitant high levels of Zn in piglets (Additional file 2: Table S2). As reported previously, this indicates an outbalanced Zn homeostasis although the expression of Zn transporters in the jejunum was regulated to reduce Zn uptake (*ZIP4*) from the gut lumen and increase the export of Zn from epithelial cells (*ZnT1*, *ZnT5*) in the Zn^high^ group. However, an increased level of Zn in intestinal tissue and the induction of metallothionein may explain our observations of improved intestinal mucosal integrity and histological changes, which are in line with other studies [12,14]. Thus, changes in absorption of water and electrolytes may counteract TGEV infection, which causes impaired intestinal epithelial barrier function and decreased uptake of micronutrients.

The paracellular epithelial barrier is highly regulated both under normal conditions and in disease. During infection, TNF-α is a mediator between the immune system and the intestinal epithelial barrier by altering tight junction proteins and their cellular localization. More specifically, the cytokine up-regulates the pore-forming protein claudin-2 [40] and redistributes the tightening protein claudin-1 [41]. This leads to an impaired barrier function which is associated with increased paracellular permeability. However, our *in vitro* work [25] and the *in vivo* study presented here did not find any up- or down-regulation of *TNF-α* after TGEV infection in any of the experimental groups. The outcome is, however, in accordance with the data set of permeability to fluorescein (P_FLU_^corr^), as paracellular permeability was not affected. The data could, therefore, be interpreted as a result of a different pathological mechanism, possibly mediated by IFN-α, rather than by TNF-α, as up-regulation of the former in Zn^low^ piglets was clearly evident.

## Conclusions

This study provides data that supplementation of the post-weaning diet with high levels of ZnO resulted in earlier and higher TGEV-specific antibody response, modulation of cytokine expression, and prevention of disruption of the intestinal barrier integrity. Our findings might also be interesting for infections with other coronaviruses like SARS-CoV, which could cause gastrointestinal symptoms and diarrhea as well.

## Methods

### Virus and cells

The TGEV strain Purdue 46-MAD (kindly provided by Dr. C. Schwegmann-Wessels, Institut für Virologie, Tierärztliche Hochschule Hannover) was used in this study. To prepare TGEV stocks, a stable mycoplasma-free swine testicle (ST) cell line supporting the growth of TGEV was used. Cells were maintained in Dulbecco’s modified Eagle’s medium (DMEM; PAN-Biotech, Aidenbach, Germany) supplemented with 10% heat-inactivated fetal calf serum (Hyclone), and 1% penicillin/streptomycin (Biochrom, Berlin, Germany) at 37°C with 5% CO_2_. Stock virus was propagated in ST cells to a titer of 1.00E + 07 plaque-forming unit (PFU)/mL.

### Animals and experimental design

German landrace piglets (n = 60) of both sexes from a TGEV-free herd (Leibniz Institut für Nutztierbiologie, Dummerstorf, Germany) were weaned at the age of 21 days and were randomly assigned to three different dietary groups (20 animals each). Diets either contained no additional ZnO (Zn^low^: 50 mg Zn/kg diet), or were supplemented with analytical grade ZnO (>98% purity) to contain 150 mg Zn/kg diet (Zn^med^) or 2,500 mg Zn/kg diet (Zn^high^). The Zn^low^ diet represents the regular feed of the animals and the Zn^low^ group, therefore, represented the control group for this experiment. At 26 days of age, animals were moved to a containment facility (Bundesinstitut für Risikobewertung, Berlin, Germany) where they were randomly allocated to 6 pens per group. The piglets were fed the respective diet *ad libitum* in a pelleted form, water was also provided *ad libitum* by nipple drinkers. The pens were of equal size (2.8 m^2^), equipped with a feeding automate with 5 feeding places and a nipple drinker. The floors were covered with rubber mate and a red light lamp was placed above to provide additional heat. Room temperature was kept at 25 ± 1°C with humidity of 50 - 60% and constant air volume exchange. The pens were thoroughly cleaned by brushing the floors and walls with consecutive flushing with lukewarm water in the mornings. Superficial flushing of the floors was additionally performed in the late afternoon. At day 28 of age, all piglets were challenged orally with 2 mL TGEV with a titer of 1.0E+07 PFU/mL. In each group, half of the piglets (n = 10 per group) were sacrificed 1 day post infection (dpi) in order to examine acute infection, since piglets reportedly display strong symptoms of gastroenteritis within 20 h post-infection [42], and the other half (n = 10 per group) were sacrificed 18 dpi to see the effect of Zn on adaptive immune response.

### Clinical follow-up and sampling

The study was approved by the local animal welfare authority (Landesamt für Gesundheit und Soziales, Berlin, Germany) under the registration number G 0116/12. Animals were clinically examined on arrival. Blood samples were collected at 0, 4, 7, 11, 14 and 18 dpi. Sera were used to determine TGEV-specific antibodies. Piglets were monitored daily for rectal temperature and body weight was recorded once weekly. Fecal scores (from 1 to 5, where 1 means watery and 5 dry and hard stool) were also recorded daily up to 12 dpi. Fecal swabs were taken before and then daily after infection for the detection of virus shedding. At necropsy (both 1 and 18 dpi), 10 – 15 cm long samples from the descending duodenum, jejunum (approximately 100 cm distal to the duodeno-jejunal flexure), ileum (distal 15 cm), spleen and jejunal mesenteric lymph nodes were taken to determine gene expression profiles. Furthermore, defined pieces of the jejunum of the same length were washed with 25 mL PBS and intestinal fluid was collected to detect adaptive immune response (sIgA). Additionally, 15 cm of mid jejunum (1 dpi) were removed, cut open, and rinsed with and transported in cooled saline solution (0.9% NaCl, 1 mM CaCl_2_). Jejunal tissue was stripped off the muscle layer and explants were mounted in Ussing chambers for electrophysiological analysis as described in detail below. Part of each intestinal tissue sample was additionally fixed in buffered 4% formalin.

### Confirmation of Zn status

Trace element status of the pigs was determined in serum and liver tissue as described previously [18]. Briefly, organs were freeze dried, incinerated and hydrolysed in concentrated hydrochloric acid. Serum samples were hydrolysed directly. Trace element concentration was determined by atomic absorption spectrometry in an AAS vario 6 spectrometer (Analytik Jena, Jena, Germany).

### Real-time quantitative RT-PCR (qRT-PCR) analysis

Total RNA was extracted from 20 mg of jejunum sample or 200 μL of serum using a Nucleo-Spin® RNA II Kit for Tissue (Macherey & Nagel, Düren, Germany) following the manufacturer’s instructions. Reverse transcription (RT) was performed using the RevertAidTM First Strand cDNA Synthesis Kit (Fermentas, St. Leon-Rot, Germany) according to the manufacturer’s instructions. PCR reactions were performed in a total volume of 25 μL in an iCycler iQ5® detection system (Bio-Rad Laboratories, München, Germany). Melt curve analysis and agarose gel electrophoresis were performed after completion of each assay to confirm specificity of the amplification. For the following target genes: Zn transporters *SLC30A1 (ZnT1)*, *SLC30A2 (ZnT2)*, *SLC30A5 (ZnT5)*, *SLC39A4 (ZIP4)*, and metallothionein *(MT1)*, quantitative real-time RT-PCR was performed using the one-step QRT-PCR master mix kit (Brilliant®II SYBR®Green, Agilent Technologies, Santa Clara, USA) as described previously [18].

TGEV genome copy numbers were quantified using a TaqMan fluorescent quantitative (q) PCR assay as previously shown [25]. Expression levels of *IFN-α*, *IL-6*, *OAS*, and *TNF-α* were calculated using the Delta-Delta-Ct-Method calculation [43]. Four commonly used reference genes (*β-2 microglobulin*, *RPL13*, *RPL19* and *SDHA*) were selected for normalization of gene expression. A mean expression value (normalization factor) for the four reference genes was calculated to enable normalization of gene expression data for all the genes of interest. Samples from Zn^med^ group were used the references for the calculations. For the expression of Zn transporters *ZnT1*, *ZnT2*, *ZnT5*, *ZIP4* and *MT1*, standard curves were generated using serial dilutions of pooled RNA (within a range from 5–200 ng/μL) from 20 samples to convert Ct values into arbitrary values. These values were then normalized using the mean values of the house-keeping genes and then used for statistical comparisons. The names of genes, primer sequences, annealing temperature, and references are listed in Additional file 1: Table S1.

### Enzyme-linked immunosorbent assay (ELISA)

Blood samples were collected on 0, 4, 7, 11, 14 and 18 dpi, and TGEV-specific IgG antibodies were determined using a commercial ELISA (INGEZIM TGEV 2.0, Ingenasa). Intestinal wash fluid was collected on 1 and 18 dpi and mucosal sIgA antibodies in the supernatants were also measured using a commercial ELISA (Pig IgA ELISA Kit, Bethyl Laboratories, Montgomery, USA) following the manufacturer’s instructions.

### Electrophysiology

Jejunal specimens were mounted in modified Ussing chambers to carry out impedance measurements as described previously [44]. In brief, one-path impedance spectroscopy was performed to determine epithelial (R^epi^) and subepithelial (R^sub^) contributions to the transepithelial resistance (TER). Resistances of bath solution without tissue as well as electrode offsets were recorded prior to each experiment and subtracted from experimental data. Preparations were allowed to equilibrate for 45 min and R^epi^ was measured as previously described [45].

As pathogen-induced secretory diarrhea is caused by excessive chloride secretion with accompanied osmotically driven water flux, forskolin (Calbiochem®, Merck, Darmstadt, Germany; final concentration 10 μM), a secretagogue agent, was added basolaterally in supplemented Ringer’s solution (113.6 mM NaCl, 5.4 mM KCl, 1.2 mM MgCl_2_, 1.2 mM CaCl_2_, 21 mM NaHCO_3_, 0.6 mM NaH_2_PO_4_, 2.4 mM Na_2_HPO_4_, 10 mM D(+)-glucose, 2.5 mM glutamine, 10 mM D(+)-mannose, 0.5 mM β-OH-butyrate, 50 mg/l piperacillin, 4 mg/l imipenem; pH 7.4 when equilibrated with carbogen) in order to trigger a chloride secretory response. For testing the sodium-coupled and therefore electrogenic glucose absorption via SGLT1, glucose (Roth, Karlsruhe, Germany; final concentration 10 mM) was added apically in glucose-free Ringer’s solution (113.6 mM NaCl, 5.4 mM KCl, 1.2 mM MgCl_2_, 1.2 mM CaCl_2_, 21 mM NaHCO_3_, 0.6 mM NaH_2_PO_4_, 2.4 mM Na_2_HPO_4_; pH 7.4 when equilibrated with carbogen). After administration of effectors, changes in short-circuit current (∆I_SC_) were recorded.

Permeability to fluorescein (332 Da) represents a measure for the paracellular leakiness/tightness of the epithelial layer and was ascertained in Ussing chamber experiments under voltage clamp conditions. After equilibrating jejunal specimens in supplemented Ringer’s solution, fluorescein (Sigma-Aldrich, St. Louis, USA) was added apically (final concentration, 50 μM). At 30 and 90 min post administration, basolateral samples were replaced with Ringer’s solution. Fluorescein concentrations were determined with a fluorometer at 525 nm (Infinite M200, Tecan, Crailsheim, Germany) and permeabilities were calculated.

### Morphometry

Formalin-fixed tissue sections were stained with hematoxylin and eosin (H&E) using standard staining protocols and analyzed using the freehand line selection tool of Image J (Rasband, ImageJ, NIH, Bethesda, Maryland; http://rsb.info.nih.gov/ij/).

The surface area of the jejunal mucosa was assessed as the ratio of mucosal-to-serosal surface area from the lengths of apical epithelial as well as muscular mucosa linings in equivalent fields of view of adjacent sections. Crypt/villus height and density were determined from five sections per piglet, each ~750 μm in width.

### Immunofluorescence staining

In order to highlight caspase-3-mediated apoptotic events in jejunal tissue, formalin-fixed tissue slices were rehydrated (xylene, increasing ethanol series), heated at 95 °C in citrate buffer (10 mM citric acid, pH 6.0) for 15 min, and then washed in phosphate-buffered saline (PBS). Tissue slices were then incubated in blocking solution (6% goat serum + 1% BSA in PBS) for 30 min at room temperature (RT) before incubation with rabbit anti-cleaved caspase-3 (Cell Signaling Technology, Cambridge, UK) at a 1:250 dilution in blocking solution for 60 min at RT. After several washings, tissue slices were incubated with goat anti-rabbit F(ab')_2_ conjugated with DyLight™488 (Jackson ImmunoResearch, Newmarket, UK) at a 1:500 dilution and 4′,6-diamidino-2-phenylindole dihydrochloride (DAPI, 1 μg/mL; Roche, Mannheim, Germany) for 60 min at RT in the dark. After washing, sections were embedded using ProTaqs Mount Fluor (Biocyc, Luckenwalde, Germany).

Images were taken with an inverted Zeiss LSM 510 META confocal laser-scanning microscope (Carl Zeiss, Jena, Germany). Digital images were processed using Fiji imaging software [46] and Zeiss LSM 510 META software.

### Statistical analyses

Calculations were performed with SPSS® Version 21 (IBM, Armonk, USA) and GraphPad Prism 5 (GraphPad Software Inc, La Jolla, USA). Two-factorial mixed models were applied to calculate residuals for all variables (fixed factor: diet, time, diet*time) and a random effect (animal). Data from gene expression were used for the one-factorial model (fixed factor: diet). F-Test was applied for fixed effects and interaction effects with subsequent LSD post hoc test. Electrophysiology and morphometric data are expressed as means ± standard error of the mean (SEM), and statistical analyses were carried out using a one-way ANOVA with Tukey HSD post hoc test. Significances are depicted as: *, *P* ≤ 0.05; **, *P* ≤ 0.01; ***, *P* ≤ 0.001; tendencies are given as 0.05 ≤ *P* ≤ 0.1.

## Abbreviations

dpi: Day post infection; H&E: Hematoxylin and eosin; IFN-α: Interferon alpha; MT1: Metallothionein-1; IL-6: Interleukin-6; OAS: Oligoadenylate synthetase; RPL13: 60S ribosomal protein L13; SDHA: Succinate dehydrogenase subunit A; ST cells: Swine testicle cells; TER: Transepithelial resistance; TGEV: Transmissible gastroenteritis virus; TNF-α: Tumor necrosis factor-alpha; ZIP4: Zn transporter SLC39A4; ZnT1: Zn transporter *SLC30A1*; ZnT2: Zn transporter *SLC30A2*; ZnT5: Zn transporter *SLC30A5.*

## Competing interests

The authors declare that they have no competing interests.

## Authors’ contributions

MB, WC and NO conceived and designed experiments. WC, SSZ, DG, RP, PJ, and ZW performed the experiments; WC, MB SSZ, RP, and ST performed statistical analyses of experimental data. WC, MB, and NO prepared the draft of the manuscript; and MB, and NO had primary responsibility for the final content. All authors critically revised the manuscript and approved the final version.

## Supplementary Material

Additional file 1: Table S1 Detailed qRT-PCR primers and conditions used in this study. Click here for file

Additional file 2: Table S2 Mean concentrations of trace elements (mg/kg fresh matter1) in liver and serum at 1 and 18 dpi. Click here for file
